# Repeated Exposure to Severely Limited Sleep Results in Distinctive and Persistent Physiological Imbalances in Rats

**DOI:** 10.1371/journal.pone.0022987

**Published:** 2011-08-11

**Authors:** Carol A. Everson, Aniko Szabo

**Affiliations:** 1 Department of Neurology, The Medical College of Wisconsin, Milwaukee, Wisconsin, United States of America; 2 Department of Population Health, The Medical College of Wisconsin, Milwaukee, Wisconsin, United States of America; Institut Pluridisciplinaire Hubert Curien, France

## Abstract

Chronic sleep disruption in laboratory rats leads to increased energy expenditure, connective tissue abnormalities, and increased weights of major organs relative to body weight. Here we report on expanded findings and the extent to which abnormalities become long-lasting, potentially permanent changes to health status after apparent recuperation from chronic sleep disruption. Rats were exposed 6 times to long periods of disrupted sleep or control conditions during 10 weeks to produce adaptations and then were permitted nearly 4 months of undisturbed sleep. Measurements were made in tissues from these groups and in preserved tissue from the experimental and control groups of an antecedent study that lacked a lengthy recuperation period. Cycles of sleep restriction resulted in energy deficiency marked by a progressive course of hyperphagia and major (15%) weight loss. Analyses of tissue composition in chronically sleep-restricted rats indicated that protein and lipid amounts in internal organs were largely spared, while adipose tissue depots appeared depleted. This suggests high metabolic demands may have preserved the size of the vital organs relative to expectations of severe energy deficiency alone. Low plasma corticosterone and leptin concentrations appear to reflect low substrate availability and diminished adiposity. After nearly 4 months of recuperation, sleep-restricted rats were consuming 20% more food and 35% more water than did comparison control rats, despite normalized weight, normalized adipocytes, and elevated plasma leptin concentrations. Plasma cholesterol levels in recuperated sleep-restricted rats were diminished relative to those of controls. The chronically increased intake of nutriments and water, along with altered negative feedback regulation and substrate use, indicate that internal processes are modified long after a severe period of prolonged and insufficient sleep has ended.

## Introduction

Repeated exposure to a deficiency of a basic need, whether food, water, oxygen, or warmth, results in physiological adaptations and phenotypic changes—modifications that are not apparent after acute deficiencies (e.g., [1], [2], [3], [4], [5], [6]). Physiological adaptations would be expected to enhance fitness and confer a survival benefit, yet physiological components of the overall response may result in changes to susceptibilities to developing medical disorders or disease. For example, obesity is considered an adaptation (e.g., [7]) that possesses a distinct survival benefit during starvation [8], [9], but also has numerous medical implications.

Inadequate sleep, in nature and in society, may be expected to be severe and recurrent during a given lifespan. We recently studied the effects of repeated exposure to 10-day periods of restricted sleep in rodents to study physiological adaptations that result from chronic limitations of sleep [10]. This approach is based on a model of cyclical weight gain and loss in humans, in which multiple cycles of starvation-refeeding in rats result in adaptive responses, such as increased lipogenesis, as well as regulatory abnormalities and medical sequelae [5]. We chose 10-day periods of sleep restriction because a single 10-day episode in rats has been shown to result in metabolic, hormonal, and immune-related signs, but to be well-tolerated [11], [12], [13], [14], [15], [16], [17], [18]. Each period between sleep-restriction cycles lasted 2 days, which previously has been shown to normalize energy expenditure and antioxidant parameters in totally sleep-deprived rats [19], [20]. Although an unfulfilled expectation, we had expected health eventually would be sustained during sleep restriction through adaptive processes. Instead, with repeated periods of sleep restriction, we observed increasingly severe, cumulative changes marked by a deep negative energy balance and adaptive changes to phenotype, including increased length of intestines and remodeling of adiposities [10].

Latent or long-lasting effects of insufficient sleep may exert injurious effects and lower resistance to disease, or otherwise alter proper development and healthy aging, in the same way that chronic restrictions of other requirements would be expected to impact health. The purpose of the present study, therefore, was to broaden understanding of the physiological characteristics that arise from chronic sleep deficiency and to determine if residual consequences remain after recuperation. To accomplish this, we analyzed the composition of preserved tissues from rats in the antecedent study of 6 cycles of sleep restriction or control conditions without extended recovery [10]. We determined the extent to which lipid, protein, or fluid are augmented or depleted by the changes to anabolism and catabolism that must underlie the excessive energy expenditure. We assessed clinical chemistry parameters and the hormones insulin, corticosterone, and leptin in preserved plasma for their diagnostic and interpretive value. The same variables were measured in a new set of rats studied for the same 6 cycles and then allowed nearly 4 months of extended recovery for detection of fresh signs or persistent abnormalities associated with prior chronic sleep loss.

## Methods

### Animals and experimental conditions

#### Ethics statement

Procedures were carried out in accordance with protocols for animal care and use approved by institutional animal care and use committees at The Medical College of Wisconsin and the Zablocki Veterans Administration Medical Center, project numbers 2560-02N and 2560-04. Subjects were adult male Sprague-Dawley rats obtained from Harlan (Madison, WI). Live animal experiments in the present study were composed of 16 rats that weighed 491 (SD 29) g and were 24 to 26 wk old at the start of the study. Preserved tissues from the antecedent study of sleep restriction without an extended recovery period were obtained from 20 rats that were 452 (SD 32) g and were 28 (SD 1) wk old at the time of study [10].

Control and experimental animals were housed under constant light conditions in rooms with ordinary ceiling lights to minimize the effects sleep deprivation may have on the amplitude and phase of the circadian rhythm (see [21]), which otherwise would be robust in the control group. Ambient temperature for rats in experimental apparatuses was maintained at 27.9 (SD 0.5)°C, within the thermoneutral zone for rats [22]. As in the antecedent study, rats were fed a diet that was isocaloric to normal laboratory chow with 12% of calories from fat and the addition of 0.26% sodium cholate.

Surgery was conducted to ensure equal treatment of rats under conditions of chronic sleep restriction or ambulation control conditions both with an extended recovery period and those of the antecedent study without an extended recovery period. Electrodes were implanted into the cranium and temporalis muscles using the procedures described for the antecedent investigation, during which electroencephalographic and electromyographic signals were obtained to determine sleep stages and wakefulness [10]. Anesthetics and analgesics administered to each rat included brief inhalation anesthesia by isoflurane followed by ketamine•HCl (100 mg/kg ip), xylazine•HCl (11 mg/kg im), and atropine sulfate (0.04 mg/kg im). Ketamine•HCl (21 mg/kg ip) supplements were used as needed to maintain the surgical plane of anesthesia. Electrodes were insulated and connected to a plug assembly affixed to the cranium. Rats recovered from surgery in experimental apparatuses on solid floors for at least 7 days before the start of a baseline period. The head plug assembly was connected to a long cable leading to a 360° swivel and counterbalanced boom assembly that permitted freedom of movement in all planes.

Schematics of the experimental apparatus designed by Rechtschaffen, Bergmann, and colleagues are shown elsewhere [23], [24]. The apparatus mainly consists of a large round platform (45 dm diam.) divided in half by a Plexiglas wall, with 1 rat housed on each side of the wall. The floor area on each side of the platform is sufficient to permit a rat to eat, groom, explore, and lie down fully to sleep. Beneath each side of the platform, a pan of shallow water approximately 2 cm deep encourages the rat to stay on the platform. The divided platform and pans are enclosed by high Plexiglas walls. The apparatus is open at the top, permitting the operation of swivels and counterbalanced boom assemblies mounted outside the enclosures. Each 6-s rotation of the housing platform induces a short ambulatory requirement because the rat is displaced, typically from a comfortable spot at the widest radius of the platform to a narrow spot that induces the rat to move to avoid stepping off the platform and into the shallow water. Specially designed food tubes allowed each rat to gnaw 1 pellet while crumbs fell into a catch receptacle, thereby allowing dependable measurement of food intake. The apparatuses were cleaned at least every other day.

During a 7-day baseline period, the platform was rotated once per hour to acquaint the rats with the movement of the housing platform.

As in the antecedent study, sleep-restricted rats experienced a cycle consisting of a 10-day sleep restriction period and a 2-day *ad libitum* sleep period, which was repeated 6 times during 72 days to permit adaptations to develop. During the sleep restriction periods, the platform was rotated for 6 s according to a schedule known to heavily fragment sleep and reduce its total accumulation without regard to sleep stage [10]. The platform rotations occupied, on average, 26% of total time across days, and the duration of the intervals between rotations varied from 1 s to more than 10 min, with a modal interval of 5 to 10 s and an average interval of 20 s. The schedule produced 2 sleep-restricted rats in each apparatus because 1 rat was housed on each side of the divided platform. During each 2-day *ad libitum* sleep period between exposures to sleep restriction, the platform was rotated only once per hour, providing opportunities for consolidated sleep.

Besides greatly fragmented sleep in sleep-restricted rats, we previously showed that the total accumulation of sleep is reduced by this technique [10]. Measurement of sleep amount in the antecedent study revealed that, during any given 10-day sleep restriction period, the percentage of time spent in non–rapid eye movement (NREM) sleep (i.e., its accumulation) is reduced from 54% during baseline to between 34 and 40% of the time. The accumulation of paradoxical sleep is reduced from 7.3% of the time during baseline to between 2.4 and 3.4% of the time. During the 2-day *ad libitum* sleep periods, NREM sleep time is consolidated and not statistically different from baseline in terms of the percentage of time. However, paradoxical sleep time rebounds and is increased by 50 to 67% above that of the baseline period [10].

The experimental conditions were matched in control animals and included surgery, daily measurement procedures, and the same total duration of ambulation requirements, except that the rotations were consolidated to permit lengthy opportunities to obtain uninterrupted sleep. The platform rotation schedule consisted of a 90-min period, during which the platform was rotated for 150 s and then was stationary for 30 s, followed by 198 min without platform rotations. This schedule was repeated 5 times per day. By these means, we have shown that NREM sleep is reduced from a baseline value of 55% of the time to between 46 and 48% of the time during a given 10-day ambulation period, while the percentage of time in paradoxical sleep does not differ from baseline. During the 2-day *ad libitum* sleep periods, time spent in NREM and paradoxical sleep does not differ from baseline [10].

Food and water intake and body weights were recorded daily. During the middle of both the 5th and 6th cycles, food and fecal waste were collected to measure caloric values, which were previously reported to rule out malabsorption and feeder waste as explanations for weight loss in rats examined in the antecedent study [10].

Rats in the antecedent study did not experience an extended recovery period; for those rats, tissue harvesting and necropsy occurred, as described below, at the conclusion of the 6 cycles of sleep restriction or ambulation control conditions. Rats in the new live animal experiments in the present study were provided a 17- to 18-wk extended recovery period after the same 6 cycles (72 days) of sleep restriction or ambulation control conditions, at which time the rats were removed from the apparatuses and placed individually in cylindrical cages (12″ diam., 12″ high). These cages were equipped with the same food tubes, water bottles, and counterbalanced boom assemblies used in the experimental apparatuses and were placed within environmental chambers under conditions of constant light and 25°C ambient temperature. Food and water intake and body weights were measured each day during the first month of extended recovery, and then every 48 hrs until Day 115 in the sleep-restricted rats, when it appeared clear that the range of body weights of this group did not differ from those of the ambulation control group. The surgically implanted head plug assemblies became detached at various points during the study, as expected; the affected individuals remained under study because the effects were transient and not serious (e.g., temporary appearance of malaise and decreased food intake).

Procedures of necropsy evaluations and tissue harvests began on Days 125 and 122 for sleep-restricted and ambulation control rats, respectively, and continued over a 7-day period, between 0900 and 1500 hr. Each rat was injected with bromodeoxyuridine (0.5 mg/kg ip) under very brief isoflurane anesthesia 90 min prior to necropsy examination to allow for future studies of cell proliferation. After this 90-min period, each rat was anesthetized by isoflurane inhalation and injected with ketamine·HCl (50 mg/kg ip), xylazine·HCl (8 mg/kg im), and atropine sulfate (0.003 mg/kg im). Once deep anesthesia was attained, a cardiac puncture was performed for blood collection and exsanguination. Blood was injected into chilled Vacutainer® tubes containing EDTA K_3_, rotated, and transferred to microcentrifuge tubes for centrifugation at 10 000 g for 10 min at 4°C.

Tissues were rapidly dissected and preserved for the present analyses and for follow-up investigations in 1 or more of the following ways: 1) enclosed in aluminum foil, snap frozen in liquid nitrogen, and sealed in small plastic bags (Whirl Pak); 2) grossed, placed in cassettes, and fixed in 10% neutral buffered formalin or 4% paraformaldehyde (adipose tissue); and 3) embedded in freezing media and fixed frozen in chilled methylbutane. The site chosen for a specimen of non-weight-bearing skeletal muscle was the ventral abdominal wall, sampled just off midline, between the rib cage and the bladder. The small intestine was dissected at the pylorus and at the junction with the cecum, laid out straight for a length measurement, and washed thrice through with saline to remove contents before preservation. Plasma and snap frozen tissues were stored at −80°C.

### Adipocyte morphometrics

Sections of paraffin-embedded adipose tissue from perirenal, omental, and epididymal sites in sleep-restricted and ambulation control rats with extended recovery were cut into 4-µm thick slices and stained with hematoxylin and eosin by the Children's Research Institute Histology Core, an affiliate of the Medical College of Wisconsin. Stained sections were coded to ensure that investigators were blind to each animal's experimental conditions. Morphometrics were performed under brightfield microscopy and digital image analysis (Olympus BX51 microscope and DP71 camera, ImagePro Plus image analysis software by Media Cybernetics, Inc., Bethesda, MD). The areas of unilocular adipocytes in each of 10 representative fields were measured. The total region sampled was approximately 0.4 mm^2^ and contained 161 to 408 adipocytes. The areas of multilocular adipocytes, identified as small, uniformly-sized lipid droplets encased within a single membrane (illustrated in [10]) also were measured. Three specimens of adipose depots were excluded from analysis because they were deemed suboptimal: 1 specimen from the mesenteric lymph node site in an ambulation control rat and 1 specimen each from the omentum and the epididymal sites in 2 sleep-restricted rats.

### Hormone and clinical chemistry assays

Rat plasma insulin and rat plasma corticosterone were measured by enzyme immunoassays (SPI-Bio, France, and Immunodiagnostic Systems Ltd, Fountain Hills, AZ, respectively). Rat plasma leptin was measured by radioimmunoassay (Millipore, St. Charles, MO). Determinations were made in duplicate. The intra-assay coefficients of variation in our hands were <4% for insulin and leptin and <10% for corticosterone. No values fell beyond the range of assay detection. The following clinical chemistry measurements were considered viable determinants in EDTA-treated plasma and were completed at the Research Animal Diagnostic Laboratory (University of Missouri, Columbia, MO): cholesterol, triglycerides, high- and low-density lipoproteins (HDL and LDL), glucose, albumin, total protein, blood urea nitrogen, creatinine, and phosphorus (Olympus AU680, Beckman-Coulter, Center Valley, PA). In addition, plasma osmolality, an important consideration in causes of thirst, was measured by the freeze-point method (Osmette, Precision Systems, Inc., Natick, MA), which is the most direct method and one that does not rely on electrolyte measurements.

### Tissue composition

Fresh frozen aliquots of liver, heart, intestine, skeletal muscle, kidney, and spleen were prepared for tissue composition analysis of fat, protein, moisture, and ash by conventional procedures (e.g., [25], [26]). Snap-frozen specimens were pulverized unthawed over dry ice. Lipids were extracted by the 2∶1 chloroform∶methanol method of Folch [27] as modified by Naito and David [28]. Chloroform extracts were evaporated overnight in aluminum micro weigh dishes under a constant-air chemical fume hood. Lipid residue was measured gravimetrically. Protein determinations were made by micro Lowry (Sigma, TP0300 and L 3540, St. Louis, MO). The moisture content of each specimen was determined by baking at 102–110°C in a dry-heat oven (Boekel Scientific, Feasterville, PA) for 20 to 24 hr. After cooling and weighing, these samples were reduced to ash in a small muffle furnace (Thermo Scientific) at 600°C for 20 to 24 hr and were then placed in a desiccant chamber to cool before weights were recorded.

### Data analysis

Values for food intake, water intake, and body weight in ambulation control and sleep-restricted rats during the six cycles and the subsequent 114 to 115 days of extended recovery, first were expressed relative to individual baseline values, then log transformed. Analyses were completed by mixed-effects models to account for different treatment conditions across time and individual variability, and to elucidate common trends. Selection of time-trend structures was guided by the Bayesian information criterion, which balances model complexity and goodness of fit. For body weight, the trend during the 6 sleep restriction cycles was modeled linearly with 1 change point, and the trend during the extended recovery period was modeled by an exponential return to an underlying linear growth rate. Both food and water intake were modeled by linear models for the separate components of sleep restriction, 2-day *ad libitum* sleep periods, and extended recovery. All 3 of these models provided a good fit both for the individual animal and the group average. Confidence intervals (CI) of the model parameters were set at 95%. The weights of organs and the size of adipocytes were compared between sleep restriction and ambulation control groups that experienced the prolonged recovery period by means of Student's *t* tests. Other variables, such as clinical chemistry parameters; hormone concentrations; or tissue components of lipid, protein, moisture, and ash, were compared by performing 3 pre-planned comparisons separately for each outcome variable in an ANOVA framework: 1) sleep-restricted compared with ambulation control conditions without extended recovery; 2) sleep-restricted without extended recovery compared with sleep restriction with extended recovery; and 3) sleep-restricted after extended recovery compared with ambulation control conditions after extended recovery. Since the components of lipid, protein, water, and ash were measured independently, their contributions did not necessarily add up to 100%. The observed values were rescaled to correct for this. The *p*-values were adjusted to control the family-wise error rate over all the comparisons within an organ at the 5% level using a bootstrap minimum *p*-value adjustment [29]. Hormone concentrations and clinical chemistry parameters were log-transformed for the analysis to improve normality.

Mixed-effects modeling was performed using the nlme 3.1–96 package of R 2.10.1. Other computations were performed in SAS 9.2 using PROC MULTTEST for the multiple comparison adjustments. Values are means (SD) if individual values composed an average and means (SE) if data first were averaged within animals and then averaged for the group.

## Results

### Body weight changes and food and water consumption

**Figure 1:**
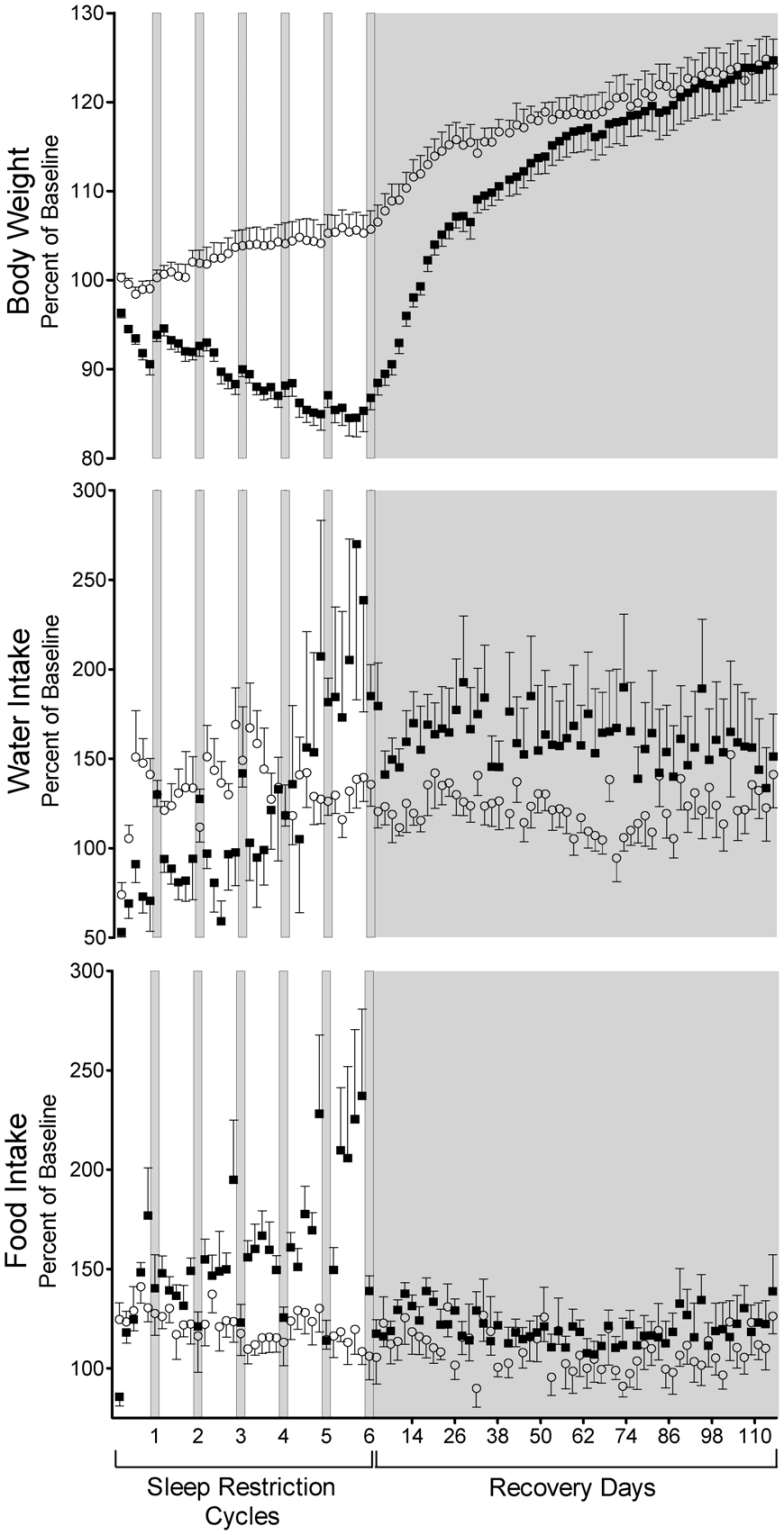
Dynamic effects on body weight, water intake, and food intake in rats resulting from 6 cycles of sleep restriction or ambulation control conditions followed by an extended recovery period. Data are expressed as a percentage change from baseline 2-day averages in sleep-restricted (▪) and ambulation control (○) rats. The first 72 days were arranged in 6 cycles, each composed of a 10-day period of sleep restriction or ambulation control (unshaded), followed by a 2-day period of *ad libitum* sleep (shaded bar). The subsequent 114 days were composed of *ad libitum* sleep conditions for recuperation (shaded area). See Results section for statistical comparison among groups and across time. Values are means (SE). *n* = 8 per group, except *n* = 7 for the sleep-restricted group during the last 2 sleep restriction cycles and during the prolonged recuperation period.

Weight loss in sleep-restricted rats was progressive across cycles of sleep restriction and averaged 15 (SD 6)% below baseline levels by the last 2 days before the extended recovery period for recuperation. This was highly significantly different from the growth of ambulation control rats during the same period (*P*<0.0001; Fig. 1). After the start of the extended recovery period, the weights of rats in both groups rebounded to the same long-term growth pattern (*P* = 0.33 for slope difference). The average body weight of the sleep-restricted group was within 95% of that of the ambulation controls by Extended Recovery Day 33 and was within 98% by Extended Recovery Day 88 (Fig. 1).

Food and water consumption during the periods of sleep restriction or ambulation control conditions before extended recovery replicated those of the antecedent study without extended recovery in overall dynamics. Peak daily food consumption based on 48-hr averages ranged from 179 to 495% of basal consumption in sleep-restricted rats of the present study. We detected initial increases in food intake at the start of both the sleep restriction and ambulation control conditions of 1.18- and 1.24-fold basal amounts, respectively, presumably due to scheduled ambulation. The positive slope of food intake during the sleep restriction periods was statistically significantly larger than that in the ambulation control group (*P*<0.0001): food intake rose 0.77% every sleep restriction day in the sleep restriction group but did not increase significantly in the ambulation control group. During the intervening 2-day *ad libitum* sleep periods, food intake in sleep-restricted rats was elevated 1.2-fold relative to baseline (95% CI: 1.08- to 1.33-fold, *P*<0.001), while food intake in ambulation control rats was at a baseline level (95% CI: 0.95- to 1.17-fold). During the extended recovery period, food intake remained elevated from basal levels by 1.2-fold in the sleep-restricted group (*P*<0.001) without an increasing or decreasing trend, while food intake was at the basal level in the ambulation control rats.

Water intake initially dropped in sleep-restricted rats by 0.58-fold (*P* = 0.0001), but then rose at an average rate of 1.12-fold every 10 days of sleep restriction (*P* = 0.013 vs. control). The 2-day *ad libitum* sleep periods in sleep-restricted rats were marked by high water intake (1.5-fold) relative to the 10-day sleep restriction periods (*P*<0.0004). High water intake continued through extended recovery in sleep-restricted rats at 1.60-fold basal amounts (95% CI: 1.36–1.88, *P*<0.0001) and without an overall time trend. This magnitude was 1.35-fold higher than that of ambulation controls during extended recovery (*P* = 0.008). In ambulation control rats, water intake was elevated (1.17-fold) during the periods of forced ambulation compared to baseline. Compared to this raised baseline, neither the 2-day nor the extended recovery periods were different, and no time trends were detected.

### Morbidity

One rat was removed from the experiment at the beginning of the 5th sleep restriction period because of a change in health status marked by lethargy, apparent weakness, and an inability to contend with platform rotations. This was the same clinical picture presented by each of 2 sleep-restricted rats that did not survive the 6th period during the antecedent study. Measurements on this animal just 24 hr prior to its removal from the formal study indicated robust food intake of 131% of baseline, reduced water intake to 47% of baseline, and loss of body weight from baseline of 12%; which should not have been lethal. Observations indicated that this rat was awake much of the time during recovery periods, when sleep was allowed *ad libitum*. The rat did not regain health during subsequent days and was euthanized. Post-mortem data were not collected from this animal.

Dermatoses began to develop on the paws of all sleep-restricted rats of this study at about the same time and at the same sites as those on sleep-restricted rats of the antecedent study [10]. Initial, small papules, typically on the main pad and in the area beneath the calcaneus bone, developed into large (8–10 mm diam.) lesions by the 6th sleep restriction period. The paws of ambulation control animals typically were free of pathology or occasionally marked by an isolated reddish spot or small papule without necrosis. The fur of sleep-restricted rats prior to extended recovery became lackluster with oiliness and denuding along the back. After prolonged recuperation, the fur had regained a normal appearance, but lesions on the hind paws of 5 of 7 sleep-restricted rats still were present as firm, circular, deep maroon-colored plaques with or without an inflamed periphery upon visual inspection. Two sleep-restricted rats each had developed an ulcerous growth with necrotic center either near the mandible (1 cm diam.) or on the back (2 cm diam.) during the extended recovery period; no such lesions were found in the ambulation control group. Necropsy evaluations of rats with extended recovery revealed healthy internal appearances, with the exception of 1 abnormal mass in the lower retroperitoneal region of an ambulation control rat that did not appear to interfere with organ functions. Neither the lengths of intestines (sleep-restricted: 110 [6 SD] cm; ambulation control: 103 [14 SD] cm) nor the weights of internal organs were significantly different between groups under conditions of extended sleep recovery.

### Adipocyte morphometrics and connective tissue signs

Adipocytes in the omentum, epididymus, and the surrounds of the mesenteric lymph nodes in sleep-restricted rats with extended recovery did not differ in size from those of corresponding ambulation control rats. Only 1 multilocular region was found in the mesentery of 1 sleep-restricted rat with extended recovery.

### Hormone and clinical chemistry results

**Figure 2:**
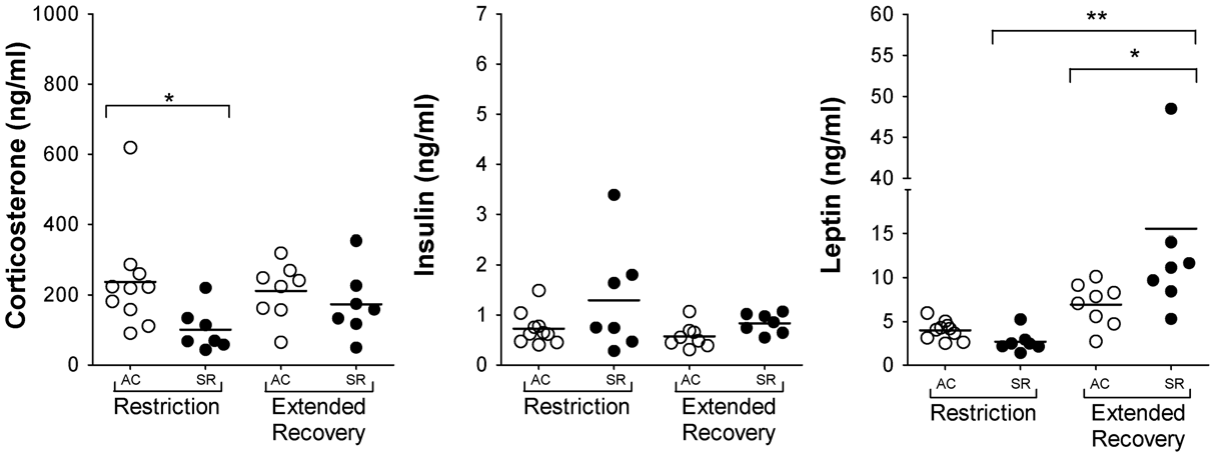
Mean basal plasma corticosterone, insulin, and leptin concentrations in individual ambulation control (○) and sleep-restricted (•) rats without and with an extended recovery period. Horizontal bars indicate group means for each treatment condition. Significance of *P<0.03 and **P<0.001 are between-group differences during sleep restriction or ambulation control treatments indicated by brackets.

Among rats that did not experience the extended recovery period, plasma corticosterone was significantly lower in sleep-restricted rats than in comparison ambulation control rats (*P* = 0.01). In contrast, sleep-restricted rats with extended recovery did not differ from corresponding ambulation control rats (Fig. 2). Plasma insulin was <1.8 ng/ml in all rats except one value of 3.4 ng/ml in a sleep-restricted rat without extended recovery that showed more advanced morbidity than did the other rats (Fig. 2). Plasma leptin was significantly higher in sleep-restricted rats with extended recovery than in corresponding ambulation control rats (*P* = 0.03; Fig. 2), despite equivalent body weights (Fig. 1). Plasma albumin was 14% lower in sleep-restricted rats without extended recovery compared with sleep-restricted rats with extended recovery (2.5 g/dl vs. 2.9 g/dl, respectively, *P* = 0.009) or with ambulation controls without extended recovery (*P* = 0.0003), in which values were considered normal (3.1 g/dl). Rats from all 4 groups were considered hyperlipidemic, having been fed an atherogenic diet to induce hypercholesterolemia [30]. Individual plasma cholesterol values ranged from 258 to 978 mg/dl among rats in all 4 groups, compared with normal plasma cholesterol values of 115 (12 SD) mg/dl for Sprague-Dawley rats [31]. Among rats that experienced extended recovery, plasma cholesterol was 31% lower in sleep-restricted rats than in controls (sleep-restricted: 449 [152 SD] mg/dl; ambulation control: 649 [173 SD] mg/dl, *P* = 0.047). The differences were further reflected by plasma LDL, determined independently and not by calculation, which was significantly lower in sleep-restricted rats with extended recovery than in control counterparts (sleep-restricted: 347 [127 SD] mg/dl; ambulation controls: 503 [126 SD] mg/dl, *P* = 0.05). No differences in the pre-planned effects were detected for the following factors measured in plasma: glucose, triglycerides, HDL, total protein, urea nitrogen, phosphorous, or creatinine. Osmolality, which represents the total molar concentration of solutes found in the blood, also did not differ by treatment.

### Tissue composition

As previously reported for sleep-restricted rats without extended recovery, the liver, heart, kidney, and small intestine each accounted for a significantly greater proportion of body weight in sleep-restricted rats compared with ambulation control rats, and spleen weight was maintained [10]. Analysis of the tissue compositions are provided in Tables 1 and 2. Compared with ambulation controls, we detected 22% less lipid in the liver (P<0.02), 12% less protein in the kidney (P<0.002), and 26% less protein in the intestine (P = 0.04) of sleep-restricted rats without extended recovery. Coincident increases in kidney and intestinal water content were observed in sleep-restricted rats compared with the ambulation controls (P = 0.02 and P = 0.04, respectively). Average values for lipid and protein contents per gram of skeletal muscle tended to be low in sleep-restricted rats, but statistical significance was not obtained. The ash content differed from that of ambulation controls in the hearts and the spleens in sleep-restricted rats without extended recovery, and in the intestines of sleep-restricted rats with extended recovery. The contribution of ash is less than 3% of the lipid free mass and is not considered further at this time. The lipid, protein, and water contents of the liver, heart, kidney, spleen, intestine, and muscle of sleep-restricted rats with extended recovery did not differ from those of ambulation controls with extended recovery.

**Table 1:** Tissue composition in chronically sleep-restricted rats and ambulation controls, with and without a lengthy recuperation period.

		*Lipid*			*Protein*			*Water*			*Ash*		
Organ/Group	Organ Weight (g)†	Per Gram (%)	Weight in Milligrams	% Organ Mass	Per Gram (%)	Weight in Milligrams	% Lipid Free Mass	Per Gram (%)	Weight in Milligrams	% Lipid Free Mass	Per Gram (%)	Weight in Milligrams	% Lipid Free Mass
**Without Extended Recovery (Not Recuperated)** a													
**Liver**													
AC	20.05 (2.99)	26.3 (3.3)	5317 (1213)	26.3 (3.3)	25.2 (2.6)	5056 (951)	34.3 (4.2)	47.3 (5.0)	9436 (1323)	64.1 (4.2)	1.2 (0.2)	240.3 (36.9)	1.7 (0.3)
SR	18.65 (2.55)	20.5 (1.9)*	3850 (816)	20.5 (1.9)*	25.8 (2.1)	4815 (768)	32.5 (2.4)	52.5 (2.2)	9759 (1136)	66.1 (2.4)	1.2 (0.1)	221.9 (22.9)	1.5 (0.1)
**Heart**													
AC	1.33 (0.10)	4.1 (0.6)	54.3 (10.0)	4.1 (0.6)	9.9 (0.9)	131.9 (16.5)	10.3 (0.9)	84.6 (1.1)	1128 (85)	88.2 (1.0)	1.5 (0.2)	19.5 (3.6)	1.5 (0.2)
SR	1.44 (0.15)	3.4 (0.3)	48.9 (6.1)	3.4 (0.3)	10.0 (1.0)	144.6 (16.9)	10.4 (1.0)	84.6 (1.0)	1222 (135)	87.6 (0.9)	2.0 (0.2)**	28.4 (4.5)**	2.0 (0.2)**
**Kidney**													
AC	2.68 (0.18)	5.0 (0.9)	135.0 (26.1)	5.0 (0.9)	15.0 (0.6)	402.9 (33.1)	15.8 (0.6)	78.2 (1.0)	2095 (137)	82.4 (0.8)	1.7 (0.2)	45.5 (6.2)	1.8 (0.2)
SR	2.68 (0.35)	5.1 (0.6)	135.1 (18.3)	5.1 (0.6)	13.2 (1.2)***	352.9 (40.1)	13.9 (1.3)**	79.7 (1.3)	2191 (271)	84.0 (1.1)*	2.0 (0.5)	55.2 (17.1)	2.1 (0.5)
**Spleen**													
AC	1.34 (0.29)	3.1 (0.6)	40.8 (12.4)	3.1 (0.6)	14.1 (2.1)	191.4 (59.0)	14.5 (2.2)	80.7 (2.2)	1077 (219)	83.3 (2.1)	2.2 (0.3)	28.8 (6.4)	2.2 (0.3)
SR	1.27 (0.29)	3.2 (0.6)	42.6 (9.6)	3.2 (0.6)	12.8 (1.0)	163.2 (37.6)	13.2 (1.0)	81.5 (1.0)	1032 (240)	84.2 (0.9)	2.5 (0.3)*	32.2 (8.7)	2.6 (0.3)*
**With Extended Recovery (Recuperated)** b													
**Liver**													
AC	31.08 (4.45)	26.4 (2.8)	8229 (1528)	26.4 (2.8)	24.1 (1.8)	7290 (728)	32.1 (1.9)	48.4 (3.2)	15216 (2736)	66.4 (1.8)	1.1 (0.1)	340.6 (55.2)	1.5 (0.1)
SR	28.35 (4.91)	25.3 (6.2)	7600 (3046)	25.3 (6.2)	26.6 (2.7)	7560 (839)	36.5 (1.7)	47.1 (4.9)	12906 (1732)	62.1 (1.7)	1.0 (0.1)	286.0 (33.4)	1.4 (0.0)
**Heart**													
AC	1.90 (0.20)	5.0 (2.4)	80.0 (14.7)	5.0 (2.4)	10.0 (0.9)	189.0 (22.7)	10.4 (1.0)	83.2 (2.4)	1592 (158)	87.5 (0.9)	1.9 (0.4)	35.9 (5.5)	2.0 (0.3)
SR	2.04 (0.40)	5.5 (2.5)	106.6 (64.8)	5.5 (2.5)	9.2 (0.5)	184.3 (39.8)	9.5 (0.8)	83.9 (2.8)	1724 (311)	88.7 (0.7)	1.4 (0.3)*	29.2 (7.3)	1.5 (0.5)*
**Kidney**													
AC	3.31 (0.33)	4.9 (0.9)	168.7 (20.7)	4.9 (0.9)	14.4 (0.9)	488.3 (52.4)	15.5 (0.8)	79.1 (1.8)	2599 (286)	82.7 (0.7)	1.6 (0.5)	56.0 (13.3)	1.7 (0.1)
SR	2.94 (0.20)	5.4 (1.3)	139.5 (17.0)	5.4 (1.3)	13.8 (0.8)	421.8 (35.6)	15.1 (0.3)	79.3 (1.1)	2333 (146)	83.3 (0.2)	1.5 (0.1)	45.6 (2.2)	1.6 (0.1)
**Spleen**													
AC	1.46 (0.57)	4.4 (1.1)	61.4 (21.1)	4.4 (1.1)	13.6 (1.1)	200.4 (80.8)	14.3 (1.1)	79.8 (1.1)	1161 (456)	83.3 (1.1)	2.3 (0.1)	34.1 (13.5)	2.4 (0.1)
SR	1.53 (0.49)	3.7 (1.4)	57.0 (26.8)	3.7 (1.4)	13.0 (1.9)	202.1 (78.6)	13.6 (2.1)	81.0 (2.3)	1229 (387)	83.9 (2.1)	2.3 (0.2)	36.6 (13.4)	2.4 (0.2)

Values are means (SD).

a N = 7–10;

b N = 6–8, except for N = 3–5 for AC and SR hearts and SR kidney weights and associated calculations of g/tissue and % organ mass. Statistical comparisons are provided for AC and SR rats without extended recovery, and AC and SR rats with extended recovery: *P<0.05, **P<0.01, ***P = 0.001 for between-group planned comparisons. Ambulation control groups are abbreviated AC, and sleep-restricted groups, SR.

† Reported for SR and AC rats without extended recovery (10).

**Table 2:** Muscle and small intestine percent composition in chronically sleep-restricted rats and ambulation controls, with and without a lengthy recuperation period.

		*Lipid*	*Protein*		*Water*		*Ash*	
Organ/Group	N	Per Gram (%)	Per Gram (%)	Lipid Free Mass (%)	Per Gram (%)	Lipid Free Mass (%)	Per Gram (%)	Lipid Free Mass (%)
**Without Extended Recovery (Not Recuperated)**								
**Intestine**								
AC	9	11.3 (2.0)	12.4 (5.3)	14.0 (3.7)	74.5 (4.0)	84.2 (1.2)	1.6 (0.2)	1.8 (0.2)
SR	8	10.3 (3.2)	9.2 (1.1)*	10.3 (1.6)*	78.9 (4.3)	87.9 (1.9)*	1.6 (0.3)	1.8 (0.3)
**Muscle**								
AC	10	9.6 (3.6)	19.4 (4.0)	21.4 (4.0)	69.6 (3.7)	77.1 (4.0)	1.4 (0.3)	1.5 (0.4)
SR	7†	6.4 (3.4)	17.0 (5.2)	18.2 (4.2)	75.1 (5.8)	79.5 (4.6)	1.4 (0.2)	1.4 (0.2)
**With Extended Recovery (Recuperated)**								
**Intestine**								
AC	8	12.7 (3.3)	10.4 (1.4)	12.0 (1.9)	75.4 (4.2)	86.4 (1.9)	1.4 (0.2)	1.6 (0.2)
SR	7	15.9 (4.1)	8.1 (1.2)	9.6 (1.2)	74.4 (3.5)	88.4 (1.2)	1.7 (0.2)*	2.0 (0.2)**
**Muscle**								
AC	8	12.5 (5.2)	22.4 (5.0)	25.7 (5.9)	64.0 (6.7)	73.1 (5.9)	1.1 (0.2)	1.3 (0.2)
SR	7	13.9 (5.1)	18.2 (2.1)	21.2 (2.8)	66.9 (5.6)	77.6 (2.7)	1.1 (0.1)	1.2 (0.1)

Values are means (SD);

† N = 5 for % lipid free mass;

* *P*<0.05,

** *P*<0.01 for two of three between-group planned comparisons: AC vs. SR without extended recovery and AC vs. SR with extended recovery.

## Discussion

The first phase of the present study replicated the failed growth and extraordinary food and water consumption observed during repeated exposure to sleep restriction without a period of extended recovery [10], thereby enabling adaptations and pathologies to be compared before and after recuperation. Prior to the extended recovery period, peak values of food intake in sleep-restricted rats typically were double and triple baseline levels, rising in a nearly cumulative manner across periods of sleep restriction, while food intake in comparison animals was stable. The degree of hyperphagia resulting from chronic sleep restriction resembles that resulting from a single prolonged period of total sleep deprivation in rats produced by the Bergmann-Rechtschaffen method [13], [14], [18], [24]. Hyperphagia also has been reported for other methods of sleep disruption, such as the pedestal or inverted flower pot technique, although not consistently (see [32]). (Discussions of methodological issues associated with the inverted flower pot technique may be found elsewhere in [32], [33], [34], [35]). Prior to the outcomes of the antecedent study, we had not predicted that a progressive and deep negative energy balance would result from repeated exposure to restricted sleep. This is because a single 10-day episode of sleep restriction in rats appears well-tolerated, and the 2-day periods of *ad libitum* sleep separating the 10-day sleep restriction periods in the present study allowed for significant sleep accumulation. In the present study, we also detected a significant 20% elevation in food intake during the 2-day *ad libitum* sleep periods, indicating adjustments and compensation for prior sleep loss, as well as a lack of any latency to the start of increased food consumption during sleep restriction.

Sleep that is disrupted and fragmented is the most plausible explanation for the metabolic effects observed. The Bergmann-Rechtschaffen paradigm has been repeatedly validated for the selective deprivation of sleep and minimization of potential confounding factors (reviewed in [36]). Our modifications of the basic paradigm to produce chronic conditions have been validated for the extent and reliability of sleep reduction and fragmentation [10]. The dramatic increases in food intake and the suppression of plasma corticosterone concentrations in sleep-restricted rats are opposite to aphagia and high corticosterone concentrations considered the hallmarks of nonspecific stress. The increased total energy expenditure appears to be a core effect of disrupted sleep and, as discussed below, some of the effects of sleep-restriction may be expected to be secondary to malnutrition and hormonal responses.

Thirst became progressively abnormal after initial increases in intake during the 2-day *ad libitum* sleep periods. Water intake was not reset to normal by prolonged recuperation, and remained persistently elevated by 35% relative to controls. A perturbation of a dipsogenic hormone cannot be ruled out, especially since the supraoptic nucleus of the hypothalamus, which is 1 of 2 brain sites of antidiuretic hormone (ADH; arginine vasopressin) synthesis, is reportedly injured by total sleep deprivation [37]. However, the increased water intake can reasonably be viewed as compensatory for the effects of chronic and excessive loads of solutes resulting from prandial intake and from the cellular demands of anabolism and catabolism (reviewed in [38], [39]). Osmoregulation by the brain is supported in the present study by findings of normal plasma osmolality. Furthermore, nonsignificant changes in plasma urea nitrogen and creatinine concentrations suggest that the waste products of metabolism were not elevated in the circulation—these are indirect indications that the effector functions of ADH were compensatory and that renal disease was not apparent. Therefore, abnormal thirst during sleep restriction appears adaptive rather than dysfunctional.

Changes to viscera resulting from sleep restriction and its associated energy deficit are not like those resulting from caloric restriction. The changes reported for internal organs of chronically sleep-restricted rats all appeared to be related to high energy production and demand [10]. Adipose tissue mass was decreased and remodeled, which included increased incidence of multilocular adipocytes shown by others to be associated with increased mitochondria biogenesis and catabolic activity [40], [41]. The small intestine was 30% longer in sleep-restricted rats than in matched control rats, consistent with an increased need for nutriment absorption [42], [43], [44], [45]. Other pathologies suggested inanition and included: loss of fattiness in connective tissues among major internal organs, hair loss, and skin lesions on the soles of the paws. However, the weights of most vital organs in sleep-restricted rats without extended recovery were increased relative to body weight. In marked contrast, during energy deficiency in rats produced by caloric restriction, the mass of internal organs is decreased [46], [47], [48], [49]. A starvation-induced loss of body weight of 17.5%, compared with a 15% loss of body weight in sleep-restricted rats, is associated with an approximate 60% loss of liver weight, 55% loss of small intestine weight, 15% loss of kidney weight, and 20% loss of heart weight [46]. By decreasing energy demands of these organs, food-restricted rodents can survive for many months at body weights 60% of those of fully fed counterparts [50], [51]. Instead, totally sleep-deprived rats will die at body weight losses averaging 22% below baseline after 16 to 27 experimental days [52].

The results indicate that lipid was diminished only in the liver by 22% and, therefore, lipid amount did not appear to be overly or selectively drawn from the vital organs. This implies the preservation of lipids as local messengers and substrate sources (reviewed in [53]). Also, proteins appeared to be spared in the liver, the heart, and the spleen; again implying their preservation for enzymatic, mechanical, and structural roles [8]. Proteins were moderately diminished in the kidney and the intestine by 12 and 22%, respectively. In marked contrast, a 17% loss of body weight in rats due to experimental caloric restriction results in protein losses of >50% in the liver, >50% in the small intestine, >17% in the kidney, and >18% in the heart [46]. We did not detect statistical differences in skeletal muscle protein and lipid, although values were lower in sleep-restricted rats compared with ambulation controls. A lack of statistical significance may have been due to a limited sample size, and a contribution by skeletal muscle to meet energy production demands during chronic sleep restriction may be especially likely considering the large proportion of body mass made up of skeletal muscle [8]. The present results on tissue composition allow the speculation that lipid stores in connective tissues may become critically exhausted under conditions of chronic sleep restriction, perhaps in the service of preserving the functions of the vital organs.

The undiminished organ masses in these hypercatabolic sleep-restricted rats are expected to reflect tissue-specific metabolic rates and energy demands at the cellular level (reviewed in [54]). The liver, kidney, heart, gastrointestinal tract, and brain normally are the largest contributors to whole body energy expenditure, with 30 to 50% of energy expenditure attributable to the liver and the intestine alone. White adipose tissue, skin, and muscle normally contribute much less to total energy demands relative to mass (reviewed in Refs. [43], [55]), and the adipose tissue and skin, in particular, showed the greatest atrophy in sleep-restricted rats. Although the etiology of skin lesions is unknown, we suspect that they are signs of inanition. This concept is supported by prior studies in which skin lesion development on totally sleep-deprived rats was less severe or of longer latency in rats fed a diet dense in calories from fat [24] or in which basal metabolism was lowered by experimental hypothyroidism [56]. The toll on skeletal muscle and connective tissues furthermore would be expected to be amplified if sleep-restricted rats were not able to double or triple their food intake. In comparison, lactating rats similarly consume 3 times more food than do nonlactating rats and lose protein mostly in muscle and skin; this protein loss is much greater if energy intake is restricted [57]. With regard to the brain, regional metabolism in both humans and rats is either unchanged or decreased by acute sleep deprivation [16], [58], [59], [60]; therefore, the brain is an unlikely source of an excessive and progressive drain on energy reserves during chronic sleep restriction. Candidate cell functions that may drive the metabolic intensity of the vital organs during sleep restriction include anabolic processes and implicate mitochondrial reactions, substrate cycling, and membrane integrity [54], [61], [62].

Plasma leptin concentrations in sleep-restricted rats without extended recovery were consistent with a large body of empirical evidence indicating that leptin is low during energy deficiency and is positively related to the amount of body fat, which appeared exhausted in these animals. In sleep-restricted rats with extended recovery, elevated leptin was associated with a persistent elevation of food intake by 20% throughout the nearly 4-month observation period, despite catch-up growth and normal adipocyte morphometrics. This is abnormal because elevated leptin is expected to suppress appetite, except in individuals with insensitivity to leptin effector functions [63]. Together, the elevated food consumption and elevated leptin concentrations point to perturbed negative feedback control over food intake. These results implicate leptin as a putative mediator in the apparent persistent elevation of energy expenditure; this mediation may occur as a result of numerous functions of leptin in affecting intermediary metabolism, neuroendocrine systems, and immunoregulation [63].

Cholesterol levels in sleep-restricted rats were opposite to those expected during an energy deficit produced by calorie restriction; food restriction leads to an increase in circulating cholesterol [64], evidently because of the mobilization of lipids from adipocytes despite decreased cholesterol biosynthesis [65]. Cholesterol and LDL levels in sleep-restricted rats with and without extended recovery are considered low, in view of 2- to 4-fold increases in the consumption of an atherogenic diet. This presumably reflects accelerated cholesterol metabolism. Drops in plasma cholesterol of 78 and 88% of control values have been found in healthy men after 4 and 5 days of sleep loss, respectively [66], although not necessarily during shorter durations of sleep restriction or poor sleep efficiency [67], [68]. A role for sleep in the synthesis of cholesterol and the proteins of lipid transport previously has been proposed on the basis of gene expression studies using microarray analyses [69], [70]. Among its many crucial roles, cholesterol functions as an antioxidant and a substrate for vitamin synthesis. LDL primarily functions to transport cholesterol from the liver to tissues that incorporate it into cell membranes. Corresponding plasma concentrations of insulin and glucose in sleep-restricted rats were normal for the most part, which suggests normal insulin sensitivity and effective management of fuel for energy.

Corticosterone concentrations in sleep-restricted rats without extended recovery were opposite to the 5-fold elevations reported for caloric deficiency produced by food restriction [71], [72], [73]. Energy deficiency is expected to stimulate the adrenal cortex to produce large quantities of glucocorticoid hormones, above all cortisol, resulting in the mobilization of proteins from essentially all cells of the body to provide substrates for conversion into glucose [74]. Putatively, a low circulating corticosterone concentration in unrecuperated sleep-restricted rats might serve to protect the vital organs by minimized lipolytic and proteolytic actions. Despite generally-held impressions, low circulating corticosterone is a consistent finding in total and partial sleep restriction in rats studied under similar experimental conditions [14], [19], [75], [76] and low or unchanged cortisol concentrations in nearly all human studies of sleep deprivation or restriction [77], [78], [79], [80], [81], [82], [83], [84], [85], [86], [87], [88], [89], [90], [91]. Small, statistically significant increases in cortisol in humans have been reported only for certain times of day in a 7-day sleep restriction protocol [92]. Exceptions in the rodent literature include studies using the inverted flowerpot or continuously rotating drum techniques, which have found increased ACTH and/or corticosterone concentrations [34], [93], [94].

Humans whose sleep is restricted for more than 24 hours show signs consistent with those of laboratory rats, suggesting comparable underlying factors and evolving adaptations and maladaptations. For example, citations of hunger in sleep-restricted humans are fairly abundant [95], [96], [97], [98], [99], as are those of decreased plasma leptin [88], [98], [100], [101], which is an early and convincing marker of nutritional or energy deficiency in humans as well as in laboratory rodents (reviewed in [63]). Fragmented sleep in humans results in increased peripheral oxygen consumption during sleep and below-normal oxygen consumption during subsequent sleep recovery [102]. As noted above, low or unchanged plasma cortisol in humans is a prevalent finding, for which the present results in rats are consistent. Findings in humans include inappropriately low TSH [88], suppressed growth hormone [103], and perhaps suppressed prolactin [104]; these changes also are observed in sleep-deprived and sleep-restricted rats and mice [14], [15], [75], [105]. Various signs of sympathetic nervous system activation are typically reported in humans [84], [88], [95], [97], [106], [107], [108], [109], [110], [111], as well as in laboratory rats [11], [18]. Sleep-deprived or sleep-restricted normal humans and laboratory rodents without increased corticosterone also share signs of increased circulating white blood cells and proinflammatory molecules [12], [76], [77], [79], [82], [90], [112], [113], [114], [115], [116], [117], [118], [119] that are known as cell injury respondents and metabolic drivers, yet without the classical clinical signs of fever or identifiable, localized inflammatory reactions.

In light of these many similarities, hypermetabolism and weight loss in sleep-restricted laboratory rodents seem at odds with results from human studies that have pointed to glucose intolerance and insulin insensitivity as consequences of sleep restriction that would promote obesity and pose a risk for type II diabetes (see Refs. [84], [120], [121], [122], [123], [124], [125], [126], [127], [128], [129]). However, body weight in humans is expected to be an extraordinarily insensitive measure of cellular functions, whereas for laboratory rats, it is much more useful because they operate closer to their maximal aerobic capacity. Despite differences in severity of sleep restriction that may underlie differences in outcomes, the effects of sleep restriction in humans may be viewed as conceptually consistent with those in rats. We hypothesize that glucose intolerance and insulin insensitivity observed in humans may be parsimoniously explained as “hunger diabetes” or “injury diabetes,” reflecting a shift in dominant fuel selection from glucose to lipids [130], as our rat data suggest. Furthermore, tests of glucose utilization in humans primarily reflect a metabolic response by skeletal muscle [130], [131], [132] and not necessarily the status of the visceral tissues; due to differential autonomic enervation and region-specific alterations. The present tissue composition analyses show differential effects of sleep restriction on muscle and the vital organs; implying muscle may be used to support metabolic demands during inadequate sleep. Comparably, a recent report of combined calorie and sleep restriction in humans cited loss of fat-free body mass and changes in substrate utilization [133].

To conclude, the present outcomes point to dynamic and fundamental physiological adjustments in response to repeated exposure to inadequate sleep. Peripheral organs and systems are largely ignored by most recent approaches to the study of sleep, which typically are brain-centered and infer that the brain is the sole recipient of benefits conferred by sleep [134], [135]. We provide evidence that signals for altered appetitive drives and negative feedback to brain foci during inadequate sleep originate in the periphery. Brain function may be considered responsive and adaptive, given the circumstances, rather than dysfunctional. The outcomes point to relatively well preserved compositions of the internal organs during repeated sleep restriction despite an energy balance tipped heavily toward catabolism. This indicates a priority for carrying out the tissue and cellular functions of vital organs at high energy costs when sleep functions are interrupted. This outcome is in marked contrast to the energy-deficient state of food restriction on every aspect examined.

The outcomes of the present studies show that recuperation from chronic sleep restriction takes a long time and that some of the physiological adaptations and potential maladaptations that arise in response to repeated exposure to sleep loss are long lasting. A period of extended recovery lasting nearly 4 months after a long bout of repeated exposure to limited sleep produced a return of an overall healthy-appearing countenance that was belied by signs of imbalance. Prominent among these signs were elevated food and water intake indicative of elevated metabolism; elevated leptin, which has many effector functions; and signs of altered substrate demands.

The present evidence prompts us to speculate that sleep enables or facilitates certain cellular processes to take place efficiently or effectively during relative immobilization because, without normal sleep, metabolic imbalance and pathological outcomes result. This speculation has its origins in long-standing ideas and theories about sleep [136], [137], [138]. While adaptive changes may increase the survivability of severe sleep restriction, changes to the mediators of the observed signs that persist during apparent recuperation could increase the likelihood of various disease processes arising, the nature of which would be expected to depend on the unique susceptibilities of the individual.
